# First‐Line Therapy in Recurrent or Metastatic Head and Neck Squamous Cell Carcinoma: A Retrospective, Multicenter, Real‐World Study

**DOI:** 10.1002/hed.28211

**Published:** 2025-06-10

**Authors:** Alizée Simon, Cyril Abdeddaim, DucTrung Nguyen, Patrice Gallet, Didier Peiffert, Aurélien Lambert, Lionnel Geoffrois

**Affiliations:** ^1^ Department of Medical Oncology Institut de Cancérologie de Lorraine Nancy France; ^2^ Faculté de médecine de Nancy Université de Lorraine Nancy France; ^3^ Department of Medical Oncology Centre Oscar Lambret Lille France; ^4^ Department of ENT ‐ Head and Neck Surgery Centre Hospitalier Regional Universitaire de Nancy Nancy France; ^5^ Department of Radiation Oncology Institut de Cancérologie de Lorraine Nancy France

**Keywords:** first‐line, head and neck cancer, immunotherapy, R/M HNSCC, real‐world

## Abstract

**Background:**

In Europe, pembrolizumab with or without chemotherapy is the recommended first‐line treatment for recurrent or metastatic head and neck squamous cell carcinoma (R/M HNSCC), with a combined positive score (CPS) ≥ 1. In France, the TPExtreme (TPEx) regimen is preferred for CPS < 1. These regimens were studied in selected populations, which may not reflect real‐world patients.

**Methods:**

We reviewed all R/M HNSCC cases treated with first‐line regimens between January 2021 and December 2023 in Nancy and Lille. Data on patient history, treatments, survival, response, and safety were collected. PFS and OS were analyzed using the Cox multivariable regression model, adjusting for the covariates age, Eastern Cooperative Oncology Group (ECOG) performance status, comorbidities, and nutritional status.

**Results:**

The analysis included 290 patients; 19% received pembrolizumab alone, 28% received pembrolizumab with chemotherapy, 19% received TPEx, and 20% received adapted regimens. PFS and OS differed significantly between groups. Median PFS and OS were respectively 2.9 and 9.4 months for pembrolizumab, 6 and 15.5 months for pembrolizumab‐chemotherapy, 5.9 and 13.7 months for TPEx, and 4.3 and 10 months for adapted regimens. Pembrolizumab alone is the safest regimen.

**Conclusion:**

This retrospective study reflects real‐world first‐line therapy for R/M HNSCC in two French cancer institutes. Pembrolizumab combined with chemotherapy is the preferred treatment option.

## 1 Introduction

Head and neck cancer incidence in France is relatively high, with approximately 16 000 new cases per year [1] and has a poor prognosis, with 50% presenting with local recurrences or metastatic disease. Historically, the first‐line treatment for recurrent or metastatic head and neck squamous cell carcinoma (R/M HNSCC) has been chemotherapy alone. Since the 2010s, the therapeutic landscape has evolved with the introduction of anti‐epidermal growth factor receptor (anti‐EGFR) therapies and immune checkpoint inhibitors. In 2020, two landmark studies changed the practice in France. The KEYNOTE‐048 (KN048) [2] demonstrated that immunotherapy alone or in combination with chemotherapy significantly improved overall survival (OS) compared to platinum‐based chemotherapy (cetuximab plus platinum and 5‐fluorouracil), especially in the subgroup of patients with a combined positive score (CPS) of 1 or more. The TPExtreme (TPEx) study [3] demonstrated a superior safety profile for docetaxel compared to 5‐fluorouracil (5‐FU) when used with platinum therapy and cetuximab, making the TPEx regimen an alternative to the standard EXTREME regimen [4]. However, the patients included in these studies were highly selected, typically with good performance status and few comorbidities, whereas patients with R/M HNSCC are often heavy smokers and alcohol consumers [5], resulting in multiple comorbidities [6]. Moreover, these patients frequently experience a decline in health status due to cancer‐related asthenia, anorexia, and malnutrition caused by the anatomical location of their tumors. Patients with heavy comorbidities, malnutrition, or an Eastern Cooperative Oncology Group (ECOG) performance status greater than 1 are excluded from clinical trials, yet they constitute a substantial portion of the real‐life population. This discrepancy highlights the need to study these patients, who often are not eligible to receive standard treatment. The CET‐MET study [7], a phase II trial comparing carboplatin, paclitaxel, and cetuximab to the EXTREME regimen, focused on identifying a new treatment option for patients with severe comorbidities and unhealthy lifestyles. It showed a favorable toxicity profile and similar progression‐free survival (PFS), making it a potential alternative to the standard of care. Additionally, the Phase IV KEYNOTE B10 study [8] showed promising results for pembrolizumab combined with carboplatin and paclitaxel, making it a potential treatment option. These studies provided several first‐line options for R/M HNSCC; in our study, we wanted to investigate which treatments are administered to these vulnerable patients in real‐world conditions.

The European Society for Medical Oncology (ESMO) recommends [9] the use of pembrolizumab for patients with a CPS ≥ 1, potentially associated with chemotherapy, and the EXTREME or TPEx regimens for patients with a CPS < 1. These recommendations apply only to patients in good health condition.

Based on this rationale, we conducted this multicenter study to assess the utilization of each treatment modality in a real‐world setting across two French cancer centers, Nancy (Institut de Cancérologie de Lorraine, ICL) and Lille (Centre Oscar Lambret, COL), which are significant recruiters of head and neck carcinoma cases. We also investigated the efficacy and safety of each treatment regimen.

## 2 Methods

### 2.1 Participants

This retrospective, multicentric study included patients treated for R/M HNSCC from January 1st 2021 to December 31th 2023, in Nancy (ICL) and Lille (COL). The patients were followed up until March 31, 2024. Adult patients (age 18 years or older) with pathologically confirmed first primary squamous cell carcinoma of the oral cavity, oropharynx, hypopharynx, larynx, sinuses, or neck lymph node metastases with unknown primary in the head and neck who had recurrent loco‐regional or metastatic disease were included; they were not eligible for local curative treatment. In ICL, we also included patients presenting with R/M HNSCC for whom palliative care only was decided as the first‐line treatment, as they were stated not eligible for standard treatment. The exclusion criteria included prior chemotherapy for HNSCC within the past 6 months and concurrent inclusion in another clinical trial.

The research committees of the ICL and COL approved the study protocol. Patients were included only if they provided written informed consent for the use of their anonymized data in scientific research. This study used the MR004 form.

### 2.2 Procedures

The patients were divided into five treatment groups. Group 1 received pembrolizumab, either 200 mg intravenously (IV) every 3 weeks or 400 mg IV every 6 weeks. Group 2 received immuno‐chemotherapy with pembrolizumab 200 mg IV and either cisplatin 80–100 mg/m^2^ IV or carboplatin AUC 5 IV, with 5‐FU 1000 mg/m^2^ IV for 4 days, every 3 weeks (KN048 protocol), in which granulocyte colony‐stimulating factor (GCSF) was not systematic. Group 3 received the TPEx regimen with cetuximab weekly (400 mg/m^2^ loading dose followed by 250 mg/m^2^ IV), combined with either 75–80 mg/m^2^ cisplatin or carboplatin AUC 5 IV and docetaxel 75–80 mg/m^2^ every 3 weeks, with systematic use of GCSF. Group 4 received other regimens, including the EXTREME and CETMET regimens, combinations of chemotherapy with or without cetuximab, and adaptations of the KN048 or KEYNOTE‐B10 regimens. A palliative care only group was analysed.

Pathologists evaluated programmed death‐ligand 1 (PD‐L1) expression using the CPS (defined as the total number of PD‐L1 positive cells, tumor cells, and immune cells, divided by the total number of tumor cells multiplied by 100). The human papillomavirus (HPV) status in oropharyngeal and oral cavity tumors was assessed by evaluating p16 expression via immunohistochemistry.

Adverse events were assessed by physicians at every consultation and were collected by reviewing every consultation report from each venue during treatment. Data were collected and graded according to the Common Terminology Criteria for Adverse Events (CTCAE) version 5.

### 2.3 Outcomes

Tumor response was evaluated using computed tomography scan (CT scan) or positron emission tomography scan (PET scan) every 2–3 months following treatment initiation until progression. Response and disease progression were assessed by a radiologist and discussed in head and neck tumor boards when necessary. In COL, the radiologist also classified the response according to the Response Evaluation Criteria in Solid Tumors (RECIST) version 1.1.

The primary endpoint was to evaluate the proportion of each treatment, including CPS, HPV status, comorbidities, tumor location, and treatment center. The secondary endpoints included OS, defined as the time from treatment initiation to death from any cause; PFS, defined as the time from treatment initiation to disease progression or death (whichever occurred first); safety; objective response rate (ORR), defined as the proportion of patients with a radiographically confirmed complete or partial response; proportion of patients progression‐free at 2, 4, and 12 months; and duration of response, defined as the time from the first documented complete or partial response to disease progression or death. The exploratory endpoint evaluated the rate of second‐line treatment received and the proportion of second‐line therapy administered.

### 2.4 Statistical Analysis

Descriptive statistics for quantitative variables were expressed as mean (±standard deviation) or median (for non‐normal distributions), and qualitative variables as percentages. Chi‐square or Fisher's exact test was used for categorical variables. For continuous variables, the Kruskal‐Wallis test was used to compare treatment groups because of the non‐normal distribution of the samples. OS, PFS, ORR, proportion of patients progression‐free at 6 and 12 months, and duration of response were evaluated in patients who received active treatment and were calculated using the Kaplan–Meier method and the log‐rank test. PFS and OS were analyzed using the Cox multivariable regression model, adjusting for the covariates age, ECOG performance status (PS), comorbidities (any chronic heart, pulmonary or liver disease), and nutritional status (defined by body mass index < 18). Comparisons of ORR were conducted using the Chi‐square test. Treatment safety was assessed by documenting the incidence and maximum severity of adverse events. Analyses were conducted using SAS v9.1 statistical software (SAS Institute, Cary, NC, USA). Statistical significance was set at *p* < 0.05.

## 3 Results

### 3.1 Study Cohort

Between January 1, 2021 and December 31, 2023, 290 patients treated for R/M HNSCC were included: 157 (54%) in ICL and 133 (46%) in COL. The mean follow‐up was 6 months (interquartile range IQR 0.5–9). The baseline demographics and disease characteristics are presented in Table 1. The median age was 65 years (range 21–94), with a significant age difference between the groups; the pembrolizumab group had the oldest population (median 70 years), and the TPEx group the youngest (median 62 years). A significant difference in treatment distribution was observed based on the PS, with a higher proportion of PS 2 patients in the pembrolizumab and adapted regimen groups. Smoking status differed significantly, with a higher percentage of current smokers in the adapted‐regimen group. Overall, 93% were former or current smokers, and 63% were former or current alcohol consumers. There were 21 (14%) HPV16‐related oral cavity or oropharyngeal cancers. Cardiovascular disease was the most common comorbidity, affecting 93 patients (32%) without influencing treatment choice. Liver disease was more frequent in patients who received adapted regimens. CPS ≥ 1 was found in 188 patients (65%).

**TABLE 1:** Characteristics of the population receiving active treatment.

	Total population^a^	Group 1 pembrolizumab	Group 2 pembro+CT	Group 3 TPEx	Group 4 adapted	*p*
Total	w290	56 (19%)	80 (28%)	56 (19%)	59 (20%)	
Location						
Nancy	157 (54%)	35 (63%)	30 (38%)	25 (45%)	29 (49%)	
Lille	133 (46%)	21 (38%)	50 (63%)	31 (55%)	30 (51%)	
Sex assigned at birth						0.06*
Men	230 (79%)	45 (80%)	58 (72.5%)	51 (91%)	45 (76%)	
Women	60 (21%)	11 (20%)	22 (27.5%)	5 (9%)	14 (24%)	
Age, years						
Median (min; max)	65 (21; 94)	70 (48; 84)	64 (33; 79)	62 (21; 76)	65 (37; 80)	< 0.001***
ECOG performance status						0.02**
0	70 (24%)	15 (27%)	27 (34%)	20 (36%)	8 (14%)	
1	122 (42%)	23 (41%)	41 (51%)	26 (46%)	29 (49%)	
2	71 (24%)	17 (30%)	11 (14%)	9 (16%)	21 (36%)	
3	26 (9%)	1 (2%)	1 (1%)	1 (2%)	1 (2%)	
4	1 (0.3%)	0	0	0	0	
Type of disease evolution at inclusion						0.38*
Metastatic^b^	128 (44%)	32 (57%)	31 (39%)	23 (41%)	26 (44%)	
De novo metastatic	44 (15%)	9 (16%)	15 (19%)	8 (14%)	8 (14%)	
Local recurrence	118 (41%)	15 (27%)	34 (42%)	25 (45%)	25 (42%)	
Previous systemic treatment^c^						
Platinum agent	97 (33%)	21 (38%)	28 (35%)	18 (32%)	23 (39%)	0.88*
Immunotherapy	6 (2%)	1 (2%)	0	4 (7%)	1 (2%)	0.004**
Primary tumor site						NA
Oral cavity	88 (30%)	13 (23%)	26 (32%)	16 (29%)	16 (27%)	
Oropharynx	71 (24%)	19 (34%)	27 (34%)	9 (16%)	12 (20%)	
Hypopharynx	59 (20%)	10 (18%)	16 (20%)	12 (21%)	15 (25%)	
Larynx	42 (14%)	8 (14%)	4 (5%)	10 (18%)	13 (22%)	
Unknown	21 (7%)	5 (9%)	4 (5%)	6 (11%)	2 (3%)	
Other	9 (3%)	1 (2%)	3 (5%)	3 (5%)	1 (2%)	
Smoking status						0.02**
Current or former	270 (93%)	52 (93%)	73 (91%)	52 (93%)	56 (95%)	
Never	19 (7%)	4 (7%)	6 (8%)	4 (7%)	3 (5%)	
Unknown	1 (< 1%)	0	1 (1%)	0	0	
Alcohol consumption						0.66*
Current or former	183 (63%)	32 (57%)	55 (69%)	37 (66%)	39 (66%)	
Never	93 (32%)	22 (39%)	23 (29%)	15 (27%)	15 (25%)	
Unknown	14 (5%)	2 (4%)	2 (2.5%)	4 (7%)	5 (8%)	
Comorbidities						
Cardiovascular	93 (32%)	23 (41%)	17 (21%)	15 (27%)	21 (36%)	0.06*
Pulmonary	62 (21%)	13 (23%)	15 (19%)	11 (20%)	18 (31%)	0.38*
Liver	23 (8%)	5 (9%)	1 (1%)	3 (5%)	12 (20%)	0.0006**
Diabetes	48 (17%)	15 (27%)	10 (13%)	5 (9%)	10 (17%)	0.052*
Previous cancer	66 (23%)	13 (23%)	12 (15%)	11 (20%)	18 (31%)	0.17*
Oropharynx or oral cavity HPV 16						0.11**
Positive	21 (14%)	5 (16%)	11 (21%)	2 (8%)	2 (7%)	
Combined positive score, CPS						
0	51 (18%)	0	0	24 (43%)	20 (34%)	
1–19	118 (41%)	18 (32%)	54 (68%)	17 (30%)	22 (37%)	
20	70 (24%)	36 (64%)	25 (31%)	3 (5%)	3 (5%)	

*Note:* Data are presented as *n* (%). Percentages might not be 100% due to rounding. Group 1, pembrolizumab alone; Group 2, pembrolizumab with chemotherapy; Group 3, TPEx regimen; Group 4, adapted regimens. For statistical tests: *: Khi‐2 test, **: Fisher exact test, ***: Kruskal‐Wallis test.

Abbreviations: CPS, combined positive score; ECOG, european cooperative oncology group; HPV, human papillomavirus; mL/min/1.73m^2^, milliliters/min/1.73m^2^; NA, not applicable because to many groups for anatomic localisation; NLR, neutrophil on lymphocyte ratio.

^a^ Total population includes patients in group 5 (palliative care).

^b^ Metastatic disease corresponds to metastatic +/− associated with loco‐regional recurrence.

^c^ Previous systemic treatment given in the neoadjuvant, adjuvant, or concurrent chemoradiotherapy curative treatment setting.

The treatment distribution was as follows: 56 (19%) patients received pembrolizumab alone, 80 (28%) received pembrolizumab with chemotherapy, 56 (19%) received the TPEx regimen, and 59 (20%) received an adapted regimen. Of the 157 patients from ICL, 38 (24%) received palliative care only at the time of diagnosis (demographics are detailed in Table 2). Carboplatin was the chosen platinum for 62 patients (77.5%) in the pembrolizumab‐chemotherapy group, and 28 patients (50%) in the TPEx group. In the adapted‐regimen group, the most frequently administered treatments were carboplatin with cetuximab (*n* = 17), carboplatin with paclitaxel (*n* = 14), and the EXTREME regimen with carboplatin (*n* = 9) (Table 3).

**TABLE 2:** Characteristics of the population receiving palliative care only.

	Group palliative care
Total	39 (13%)
Location	
Nancy	38 (97%)
Lille	1 (3%)
Sex assigned at birth	
Men	31 (79%)
Women	8 (21%)
Age, years	
Median (min; max)	65 (51; 94)
ECOG performance status	
0	0
1	3 (8%)
2	13 (33%)
3	22 (56%)
4	1 (2.5%)
Type of disease evolution at inclusion	
Metastatic	16 (41%)
De novo metastatic	4 (10%)
Loco recurrence	19 (49%)
Previous systemic treatment	
Platinum agent	7 (18%)
Immunotherapy	0
Primary tumor site	
Oral cavity	17 (44%)
Oropharynx	4 (10%)
Hypopharynx	6 (15%)
Larynx	7 (18%)
Unknown	4 (10%)
Other	1 (3%)
Smoking status	
Current or former	37 (95%)
Never	2 (5%)
Unknown	0
Alcohol consumption	
Current or former	20 (51%)
Never	18 (46%)
Unknown	1 (3%)
Comorbidities	
Cardiovascular	17 (44%)
Pulmonary	5 (13%)
Liver	2 (5%)
Diabetes	8 (21%)
Previous cancer	12 (31%)
Oropharynx or oral cavity HPV‐induced	
Positive	1 (5%)
Combined positive score	
0	7 (18%)
1–19	7 (18%)
20	3 (8%)

*Note:* Data are *n* (%). Percentages might not total 100% due to rounding.

Abbreviations: ECOG, European Cooperative Oncology Group; HPV, human papillomavirus.

**TABLE 3:** Characteristics of the combined treatments.

Treatment regimen	Group 2 pembro+CT	Group 3 TPEx	Group 4 adapted
Carboplatin regimen	62 (77.5%)	28 (50%)	
Cisplatin regimen	18 (22.5%)	28 (50%)	
Carboplatin, paclitaxel, and cetuximab			14 (24%)
Carboplatin and 5FU			1 (2%)
Carboplatin and cetuximab			17 (29%)
Paclitaxel and cetuximab			3 (5%)
EXTREME regimen (carboplatin, 5FU and cetuximab)			9 (15%)
Platinum and pembrolizumab			
Carboplatin regimen			4 (7%)
Cisplatin regimen			1 (2%)
KN‐B10 regimen (carboplatin, paclitaxel and pembrolizumab)			2 (4%)

*Note:* Data are *n* (%). Percentages might not total 100% due to rounding. Group 2, pembrolizumab with chemotherapy, Group 3, TPEx regimen, Group 4, adapted regimens.

Abbreviations: 5FU, 5 fluorouracil; KB10, Keynote‐B10.

### 3.2 Effectiveness

ORR was 18% in the pembrolizumab group (10 patients, with 1 complete response at 2–12 months), 57.5% in the pembrolizumab‐chemotherapy group (46 patients, with 1 complete response at 2–12 months), 59% in the TPEx group (33 patients), and 39% in the adapted regimens group (23 patients, 2 complete responses at 2 and 4 months). The median duration of response was 3.5 months [IQR, 3.1–9] for pembrolizumab, 5.9 [2.4–8.7] for pembrolizumab‐chemotherapy, 4.6 [3–6.9] for TPEx, and 4.4 [2.7–6.3] for adapted regimens (Table 4).

**TABLE 4:** Response rate and duration of response.

	Total	Group 1 pembrolizumab	Group 2 pembro+CT	Group 3 TPEx	Group 4 adapted	*p*
Responding at 2 months	103 (41%)	10 (18%)	41 (51%)	30 (54%)	22 (37%)	
Responding at 4 months	51 (20%)	4 (7%)	23 (29%)	13 (23%)	11 (19%)	
Responding at 12 months	11 (4%)	1 (2%)	7 (9%)	3 (5%)	0	
Best overall response						
Partial response	96 (38%)	8 (14%)	40 (50%)	27 (48%)	21 (36%)	
Complete response	16 (6%)	2 (4%)	6 (7.5%)	6 (11%)	2 (3%)	
Total	112 (45%)	10 (18%)	46 (57.5%)	33 (59%)	23 (39%)	
Duration of response						
Median (IQR) in months		3.5 (3.1–9)	5.9 (2.4–8.7)	4.6 (3–6.9)	4.4 (2.7–6.3)	0.98

*Note:* Data are presented as *n* (%) or median (IQR). Percentages might not total 100% due to rounding. Group 1, pembrolizumab alone; Group 2, pembrolizumab with chemotherapy; Group 3, TPEx regimen; Group 4, adapted regimens.

Abbreviation: IQR, interquartile range.

**FIGURE 1:**
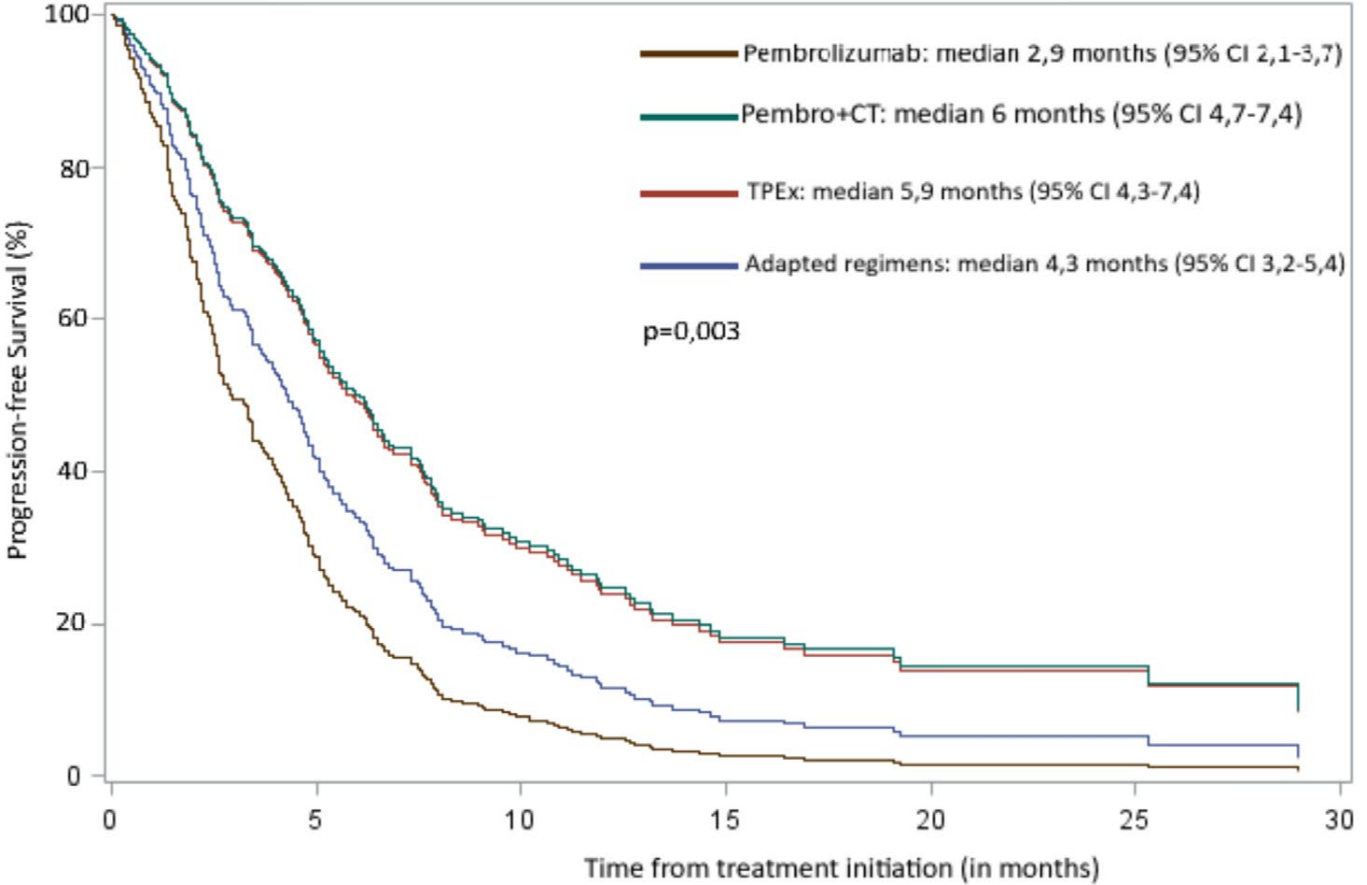
Kaplan–Meier estimates of progression free survival. 95% CI, 95% confidence interval; Pembro+CT, pembrolizumab with chemotherapy; TPEx, TPExtreme chemotherapy regimen. [Color figure can be viewed at wileyonlinelibrary.com]

By using COX multivariate analysis while adjusting for the covariates age, PS, comorbidities, and nutritional status, we identified independent factors impacting the PFS as follows: age (HR = 0.96, [95% CI 0.95–0.98], *p* < 0.001); PS (HR = 1.49, [1.09–2.04], *p* = 0.013), and the type of treatment compared to pembrolizumab alone (*p* < 0.0001), pembrolizumab with chemotherapy (HR = 0.42, [0.29–0.63]); TPEx regimen (HR = 0.44, [0.29–0.67]), and adapted regimen (HR = 0.69, [0.47–1.02]). PFS differed significantly between groups, with a median PFS of 2.9 months [95% CI 2.1–3.7] for pembrolizumab, 6 [4.7–7.4] for pembrolizumab‐chemotherapy, 5.9 [4.3–7.4] for TPEx, and 4.3 [3.2–5.4] for adapted regimens (Figure 1).

By the same way, we identified independent factors impacting the OS as follows: age (HR = 0.96, [95% CI 0.95–0.98], *p* < 0.001); PS (HR = 1.98, [1.39–2.82], *p* = 0.0002), and the type of treatment compared to pembrolizumab alone (*p* = 0.0064), pembrolizumab with chemotherapy (HR = 0.52, [0.33–0.83]); TPEx regimen (HR = 0.62, [0.38–1.01]), and adapted regimen (HR = 1.0, [0.64–1.56]).

**FIGURE 2:**
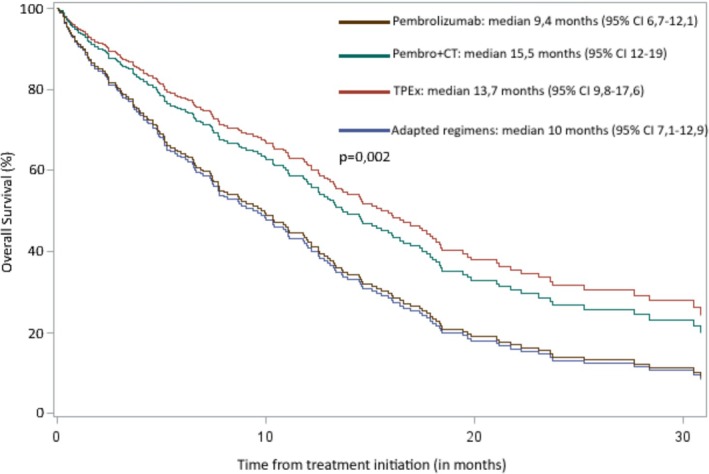
Kaplan–Meier estimates of overall survival. 95% CI, 95% confidence interval; Pembro+CT, pembrolizumab with chemotherapy; TPEx, TPExtreme chemotherapy regimen. [Color figure can be viewed at wileyonlinelibrary.com]

OS also showed significant differences with a median OS of 9.4 months [95% CI 6.7–12.1] for pembrolizumab, 15.5 [12–19] for pembrolizumab combined with chemotherapy, 13.7 [9.8–17.6] for TPEx, and 10 [7.1–12.9] for adapted regimens (Figure 2). Median OS for palliative care was 2.4 months [95% CI, 1.4–2.8].

### 3.3 Toxicity

The toxicity data are summarized in Table 5. Pembrolizumab combined with chemotherapy and TPEx showed similar toxicity profiles, with 100% of patients experiencing adverse events of any grade (59% grade 3 or worse) in the TPEx group and 95% (56% grade 3 or worse) in the pembrolizumab combined with chemotherapy group. Pembrolizumab and adapted regimens had more favorable safety profiles, especially pembrolizumab alone, with only 20% of grade 3 or worse events. One treatment‐related death occurred in each group: myositis (pembrolizumab), infectious pneumonia (pembrolizumab combined with chemotherapy), interstitial pneumonia (TPEx), and febrile neutropenia (paclitaxel‐cetuximab). The most common adverse events of any grade were fatigue, anemia, and skin disorders. The most frequent grade 3 or higher events were neutropenia, anemia, and infection. Pembrolizumab is associated with a high risk (≥ 25%) of dysthyroidism, and chemotherapy is associated with a high risk of blood disorders, nausea, diarrhea, infection, and mucositis.

**TABLE 5:** Adverse events.

	Group 1 (56) pembrolizumab	Group 2 (80) pembro+CT	Group 3 (56) TPEx	Group 4 (59) adapted	Total (251)
Anemia					
Any grade	0	57 (71%)	24 (43%)	39 (66%)	120 (48%)
Grade 3–4	0	16 (20%)	5 (9%)	4 (7%)	25 (10%)
Thrombopenia					
Any grade	0	23 (29%)	6 (11%)	11 (19%)	40 (16%)
Grade 3–4	0	8 (10%)	2 (4%)	4 (7%)	14 (6%)
Neutropenia					
Any grade	2 (4%)	22 (28%)	6 (11%)	22 (37%)	52 (21%)
Grade 3–4	1 (2%)	15 (19%)	4 (7%)	14 (24%)	34 (14%)
Febrile neutropenia					
Grade 3–5	1 (2%)	3 (4%)	1 (2%)	5 (8%)	10 (4%)
Peripheral sensory neuropathy					
Any grade	0	5 (6%)	9 (16%)	12 (20%)	26 (10%)
Grade 3–4	0	0	1 (2%)	0	1 (0.5%)
Allergic reaction					
Any grade	0	1 (1%)	6 (11%)	3 (5%)	10 (4%)
Grade 3–4	0	1 (1%)	3 (5%)	1 (2%)	5 (2%)
Diarrhea					
Any grade	8 (14%)	21 (26%)	25 (45%)	12 (20%)	66 (26%)
Grade 3–4	2 (4%)	3 (4%)	5 (9%)	0	10 (4%)
Nausea					
Any grade	2 (4%)	28 (35%)	16 (29%)	12 (20%)	58 (23%)
Grade 3–4	0	5 (6%)	0	1 (2%)	6 (2%)
Vomiting					
Any grade	0	14 (18%)	7 (13%)	7 (12%)	28 (11%)
Grade 3–4	0	3 (4%)	0	0	3 (1%)
Fatigue					
Any grade	21 (38%)	46 (58%)	36 (64%)	34 (58%)	137 (55%)
Grade 3–4	4 (7%)	3 (4%)	5 (9%)	2 (3%)	14 (6%)
Hypokalaemia					
Any grade	3 (5%)	10 (13%)	12 (21%)	10 (17%)	35 (14%)
Grade 3–4	0	1 (1%)	1 (2%)	0	2 (1%)
Hypomagnesaemia					
Any grade	0	5 (6%)	25 (45%)	25 (42%)	55 (22%)
Grade 3–4	0	0	1 (2%)	2 (3%)	3 (1%)
Skin disorder (dermatitis, rash)					
Any grade	11 (20%)	24 (30%)	45 (80%)	35 (59%)	115 (46%)
Grade 3–4	0	1 (1%)	6 (11%)	0	7 (3%)
Decreased appetite					
Any grade	5 (9%)	11 (14%)	9 (16%)	10 (17%)	35 (14%)
Grade 3–4	2 (4%)	1 (1%)	1 (2%)	1 (2%)	5 (2%)
Infection (any type)					
Any grade	4 (7%)	20 (25%)	11 (20%)	10 (17%)	45 (18%)
Grade 3–5	3 (5%)	18 (22.5%)	11 (20%)	10 (17%)	40 (16%)
Mucositis oral					
Any grade	0	22 (28%)	19 (34%)	12 (20%)	53 (21%)
Grade 3–4	0	3 (4%)	1 (2%)	1 (2%)	5 (2%)
Dysthyroidism					
Grade 1–2	14 (25%)	20 (25%)	2 (4%)	1 (2%)	37 (15%)
Respiratory disorder (pneumonitis)					
Any grade	1 (2%)	0	1 (2%)	0	2 (1%)
Grade 3–5	1 (2%)	0	1 (2%)	0	2 (< 1%)
Total					
Any grade	41 (73%)	76 (95%)	56 (100%)	56 (95%)	229 (91%)
Grade 3–4	11 (20%)	45 (56%)	33 (59%)	29 (49%)	118 (47%)
Grade 5	1 (2%)	1 (1%)	1 (2%)	1 (2%)	4 (2%)

*Note:* Data are *n* (%). Percentages might not total 100% due to rounding. The data presented include adverse events of any grade occurring in ≥ 15% of patients and grade 3 or higher adverse events occurring in ≥ 5% of patients. Group 1, pembrolizumab alone; Group 2, pembrolizumab with chemotherapy; Group 3, TPEx regimen; Group 4, adapted regimens.

### 3.4 Second‐Line

We investigated second‐line treatments for the patients who received them (Data S1). 51%–61% of patients in each group received a second‐line treatment, mostly nivolumab, for patients who did not receive first‐line immunotherapy, and taxanes with cetuximab, with or without platinum, for patients who did.

## 4 Discussion

Our findings indicate that ESMO recommendations are generally followed in a real‐world setting in these two French cancer institutes. Pembrolizumab, alone or in combination with chemotherapy, is favored for patients with a CPS ≥ 1, while the TPEx regimen remains common for patients with a CPS < 1. The survival outcomes in our study seem superior for pembrolizumab combined with chemotherapy and TPEx. The safety profile of pembrolizumab supports its use as it is associated with fewer adverse events. The safety profiles of TPEx and pembrolizumab combined with chemotherapy are similar, making it difficult to favor one over the other for patients who can receive both. However, adapted regimens showed a better safety profile than triple therapies, suggesting that they may be preferred for patients with poor general health status and comorbidities who are not eligible for pembrolizumab.

Our findings showed survival outcomes similar to those of Keynote‐048 for pembrolizumab combined with chemotherapy, with a median OS of 15.5 months and a PFS of 6 months in our study, compared to 13.6 months OS and 5.0 months PFS for the CPS ≥ 1 subgroup in Keynote‐048. Similarly, pembrolizumab in our study had an OS of 9.4 months and a PFS of 2.9 months, compared to 12.3 months OS and 3.2 months PFS reported by Keynote‐048. Regarding the TPEx regimen, our study reported a median OS of 13.7 months and a PFS of 5.9 months, compared to 14.5 months OS and 6.0 months PFS in the original TPEx study. Although the OS for TPEx in our study was slightly lower, the outcomes were generally similar, demonstrating the consistency of this treatment in the real‐world setting. This difference could be attributed to the TPEx study limiting cisplatin as platinum therapy in the first cycle, while 50% of our patients received carboplatin. Our study shows that real‐world outcomes are comparable to those of randomized trials, which is reassuring for their application in clinical practice.

Survival outcomes suggest that triple therapies may be superior; however, the age and PS bias must be considered. In our study, elderly and fragile patients were more likely to receive pembrolizumab or adapted regimens. Consequently, we cannot definitively conclude that pembrolizumab combined with chemotherapy and TPEx is superior due to patient population disparities between groups, constituting a bias. We performed a Cox analysis to mitigate this bias; however, a second bias persists—the relatively small sample size limits robust comparisons across all four groups, as we did not perform direct pairwise comparisons but analyzed all groups collectively.

Our study provides important insights into the high toxicity associated with triple treatment regimens in a real‐world setting. This emphasizes the need to identify suitable treatments for frail patients, who are often excluded from clinical trials. Real‐world studies such as ours are essential to evaluate the efficacy and safety of recommended treatments in diverse populations. This study also advocates a thorough assessment of general health status before initiating a triplet regimen, given the 100% incidence of adverse events. This finding reminds us of the need to balance the benefits and drawbacks of palliative treatments that aim to prolong life and enhance its quality. We collected data among patients ineligible for active treatment who received palliative care only, representing approximately 25% of the population in Nancy. The associated toxicity could worsen their general health with a minimal chance of prolonging their lives.

Our findings showed that approximately 50% of patients experienced grade 3 or worse adverse events in both the TPEx and pembrolizumab combined with chemotherapy groups. These results differ from those of the TPEx study, which reported 83% grade 3 or worse adverse events, and Keynote‐048, which reported 85%. This difference could be attributed to the general improvement in managing these regimens, as clinicians have gained experience in handling their toxicity for several years since the initial studies were published. However, it could also be explained by a potential underreporting of adverse events in our study, which was based on retrospectively collected real‐world data, compared to clinical trials.

The low overall response rate of pembrolizumab alone demonstrates the need to improve the antitumor immune response. One approach could be to identify new predictive factors for selecting patients who are likely to respond to immunotherapy, as CPS alone may not be an effective marker. Another strategy could be to combine immunotherapies, such as the CheckMate‐651 combined anti‐CTLA4 ipilimumab with anti‐PDL1 nivolumab, but this failed to improve survival [10]. The scientific community has explored new treatment combinations with immunotherapy. Current clinical trials have evaluated the combination of anti‐PDL1 and anti‐EGFR, such as Sacco et al. [11] investigating pembrolizumab with cetuximab or Chung et al. [12] investigating nivolumab with cetuximab in a second‐line setting. Innovative therapies, such as petosemtamab, a bispecific antibody, are also being investigated in association with immunotherapy, as presented at ASCO 2024 [13]. In Northern Europe and North America, HPV‐related cancers are the most frequent type of head and neck cancers [14]; however, our study indicates that most cases in these two French cancer institutes are still non‐HPV‐related. Further research is needed to improve the survival and quality of life of this population. A recent meta‐analysis suggested better outcomes for HPV‐negative patients treated with the combination of cetuximab and anti‐PDL1, also presented at the ASCO 2024 [15]. Another promising approach is to combine immunotherapy with taxanes. We found that pembrolizumab with 5FU and platinum was effective, as was cetuximab associated with taxane and platinum. However, the combination of immunotherapy with platinum and taxanes in the first‐line setting has not yet been tested in a randomized phase III study. A regimen that combines immunotherapy with platinum and taxanes could be a promising approach for future research.

## 5 Conclusion

Our real‐world study enabled us to describe the treatment regimens mostly used in our two French cancer institutes for R/M HNSCC. The most frequently administered treatment is pembrolizumab combined with chemotherapy, followed by pembrolizumab alone and the TPEx regimen. Some patients received adapted regimens owing to comorbidities or poor general health status. The survival outcomes in this study are consistent with those reported in clinical trials. Safety remains a concern, as triple therapies are associated with a nearly 100% incidence of adverse events and a 50% incidence of grade 3 or worse events. Further research is needed to identify optimal treatment modalities to improve survival outcomes and safety.

## Author Contributions

Conception and design: Alizée Simon and Lionnel Geoffrois. Provision of study material or patients: Alizée Simon, Lionnel Geoffrois, and Cyril Abdeddaim. Collection and assembly of data: Alizée Simon. Data analysis and interpretation: Alizée Simon, Lionnel Geoffrois, Cyril Abdeddaim, and Duc Trung Nguyen. Manuscript writing: Alizée Simon, Lionnel Geoffrois, Cyril Abdeddaim, Duc Trung Nguyen, Aurélien Lambert, Didier Peiffert, and Patrice Gallet. Final approval of manuscript: All authors. Accountable for all aspects of the work: All authors.

## Conflicts of Interest

The authors declare no conflicts of interest.

## Supporting information


**Data S1.** hed28211‐sup‐0001‐supinfo.

## Data Availability

The data that support the findings of this study are available on request from the corresponding author. The data are not publicly available due to privacy or ethical restrictions.
