# Supplementary material for: First‐Line Therapy in Recurrent or Metastatic Head and Neck Squamous Cell Carcinoma: A Retrospective, Multicenter, Real‐World Study
Source: Head Neck. 2025 Jun 10;47(11):2939–49. doi: 10.1002/hed.28211 (PMC12541674; doi:10.1002/hed.28211)
Supplement: Supplementary file 1 — Data S1. hed28211‐sup‐0001‐supinfo. [file HED-47-2939-s001.docx]

## **SUPPLEMENTARY FILE**

Table 6: Second line treatment

| Second line treatment modality | Group 1  Pembrolizumab | Group 2  Pembro + CT | Group 3  TPEx | Group 4  Adapted | Total |
| --- | --- | --- | --- | --- | --- |
| Received | 27 (48%) | 43 (54%) | 34 (61%) | 30 (51%) | 138 (55%) |
| Nivolumab | 0 | 0 | 27 (48%) | 22 (37%) | 49 (20%) |
| Paclitaxel and cetuximab | 1 (2%) | 17 (21%) | 1 (2%) | 1 (2%) | 20 (8%) |
| TPEx regimen: Carboplatin, docetaxel and cetuximab | 9 (16%) | 4 (5%) | 0 | 0 | 13 (5%) |
| Carboplatin and paclitaxel | 4 (7%) | 4 (5%) | 3 (5%) | 2 (3%) | 13 (5%) |
| Paclitaxel | 1 (2%) | 7 (9%) | 0 | 1 (2%) | 9 (4%) |
| Methotrexate | 4 (7%) | 1 (1%) | 2 (4%) | 3 (5%) | 10 (4%) |
| Clinical trial | 1 (2%) | 7 (9%) | 0 | 0 | 8 (3%) |
| Carboplatin | 2 (4%) | 0 | 0 | 0 | 6 (2%) |
| Carboplatin, paclitaxel and cetuximab | 2 (4%) | 3 (4%) | 0 | 1 (2%) | 6 (2%) |
| Carboplatin and cetuximab | 2 (4%) | 0 | 1 (2%) | 0 | 3 (1%) |
| EXTREME regimen: Carboplatin, 5FU and cetuximab | 1 (2%) | 0 | 0 | 0 | 1 (0.5%) |

*Data are n (%). Percentages might not total 100% due to rounding.
Group 1: pembrolizumab alone, group 2: pembrolizumab with chemotherapy, group 3: TPEx regimen, group 4: adapted regimens*

Table 7: Comparisons between our study and pivotal trials, data provided for reference.

|  | OS | PFS | DR | ORR | Toxicity | ≥G3 |
| --- | --- | --- | --- | --- | --- | --- |
| Pembrolizumab  Group 1  KN048 | 12.2m  12.3m | 2.9m  3.2m | 3.5m  22.6m | 18%  19% | 73%  NC | 20%  55% |
| Pembro-chimio  Group 2  KN048 | 16.1m   - 1. m | 5.9m  5.0m | 5.9m  7.1m | 57.5%  38% | 95%  NC | 56%  85% |
| TPEx  Group 3  TPEx | 11.9m  14.5m | 5.7m  6.0m | 4.6m  NC | 59%  57% | 100%  NC | 59%  81% |
| Adapted regimens  Group 4 | 8m | 4.7m | 4.4m | 39% | 95% | 49% |

*Abbreviations: Pembro-chemo: pembrolizumab combined with chemotherapy, KN08: Keynote-048 trial, TPEx: TPExtreme trial, OS: Overall Survival, PFS: Progression-Free Survival, DR: Duration of Response, ORR: Objective Response Rate, Toxicity: all-grade toxicity, ≥G3: grade 3 or higher toxicity, NC: not known, m: months*


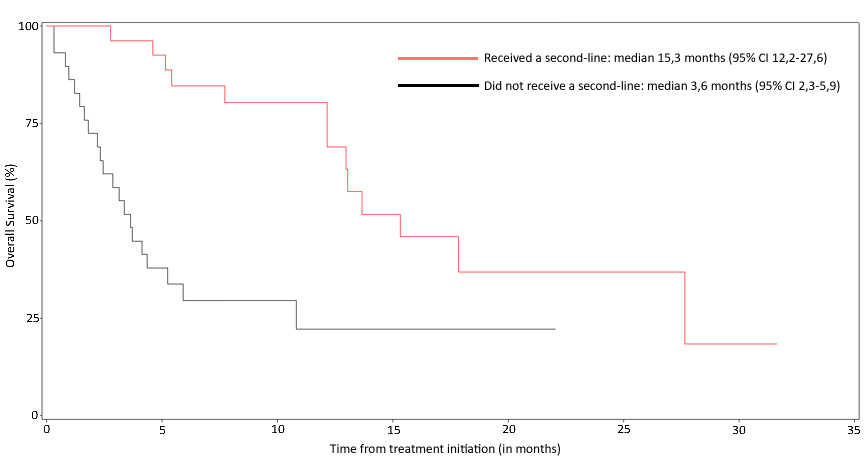


Figure 3: Kaplan-Meier estimates of overall survival for group 1 comparing comparing patients who received a second line treatment and those who did not

*Abbreviations: 95% CI: 95% confidence interval*


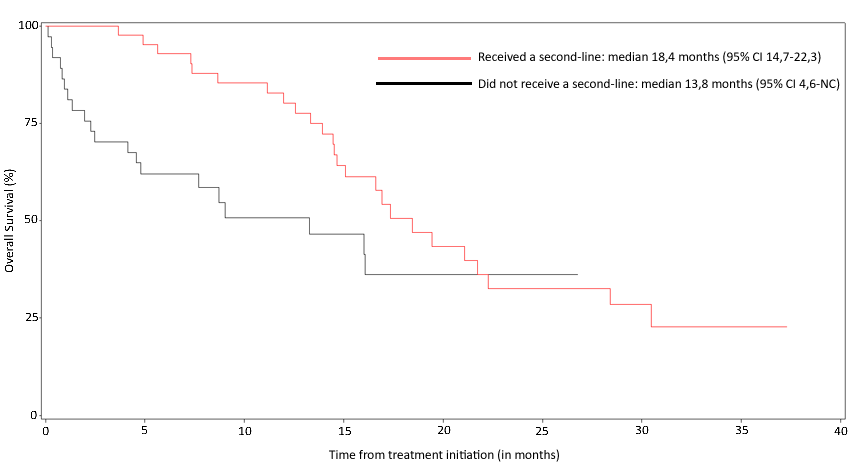


Figure 4: Kaplan-Meier estimates of overall survival for group 2 comparing comparing patients who received a second line treatment and those who did not

*Abbreviations: 95% CI: 95% confidence interval, NC: not calculable*
